# ﻿*Yersinochloanghiana*, a new species (Poaceae, Bambusoideae, Bambuseae) from southern Vietnam

**DOI:** 10.3897/phytokeys.224.101201

**Published:** 2023-04-07

**Authors:** Tran Thai Vinh, Nong Van Duy, Hoang Thanh Truong, Tran Van Tien

**Affiliations:** 1 Taynguyen Institute for Scientific Research, Vietnam Academy of Science and Technology, Dalat, Vietnam Taynguyen Institute for Scientific Research, Vietnam Academy of Science and Technology Dalat Vietnam; 2 Graduate University of Science and Technology, Vietnam Academy of Science and Technology, Hanoi, Vietnam Graduate University of Science and Technology Hanoi Vietnam; 3 Vietnam Forest Science Institute of Central Highlands and South of Central Vietnam, Vietnam Academy of Forest Science, Dalat, Lam Dong Province, Vietnam Vietnam Forest Science Institute of Central Highlands and South of Central Vietnam, Vietnam Academy of Forest Science Dalat Vietnam; 4 Dalat University, Dalat, Lam Dong Province, Vietnam Dalat University Dalat Vietnam

**Keywords:** new species, Vietnam, *Yersinochloa*

## Abstract

*Yersinochloanghiana***sp. nov.** from Vietnam is described and illustrated. It is found from southern Vietnam, where it occurs at an elevation of 1130 m in Braian Mountain, Di Linh District, Lam Dong Province. This new species is distinguished from a similar species, *Yersinochloadalatensis*, by culm nodes with a thick swollen patella, culm leaf blades erect, auricles conspicuous, margins bearing long hairs, palea dorsal view showing rachilla extension and rudimentary floret at the apex and lodicules bifid at the base.

## ﻿Introduction

Recently, the morphology of clambering or scrambling bamboo genera in the tribe Bambuseae Kunth ex Nees, subtribe Bambusinae J. S. Presl have been reviewed in depth (Nguyen and Tran 2012; Nguyen et al. 2013; Nguyen and Tran 2016). Seven distinct clambering or scrambling bamboo genera in Asia are currently recognised: 1) *Maclurochloa* K.M. Wong (Wong 1993); 2) *Soejatmia* K.M. Wong from the Malay Peninsula (Wong 1993); 3) *Neololeba* Widjaja from the Philippines, central and eastern Indonesian islands, New Guinea and Queensland (Widjaja 1997); 4) *Mullerochloa* K.M. Wong from northeast Australia (Wong 2005); 5) *Nianhochloa* H.N. Nguyen & V.T. Tran from southern Vietnam (Nguyen and Tran 2012); 6) *Cochinchinochloa* H.N. Nguyen & V.T. Tran from southern Vietnam (Nguyen et al. 2013) and 7) *Yersinochloa* H.N. Nguyen & V.T. Tran from southern Vietnam (Nguyen and Tran 2016). The systematics of these clambering or scrambling bamboo genera have traditionally been given by vegetative and productive characters (Nguyen and Tran 2016). One of these, *Yersinochloa* H.N. Nguyen & V.T. Tran of Vietnam, is specially characterised by pseudo-spikelets with only one perfect floret, unkeeled palea and anther apices with tiny spines (Nguyen and Tran 2016).

**Figure 1.**
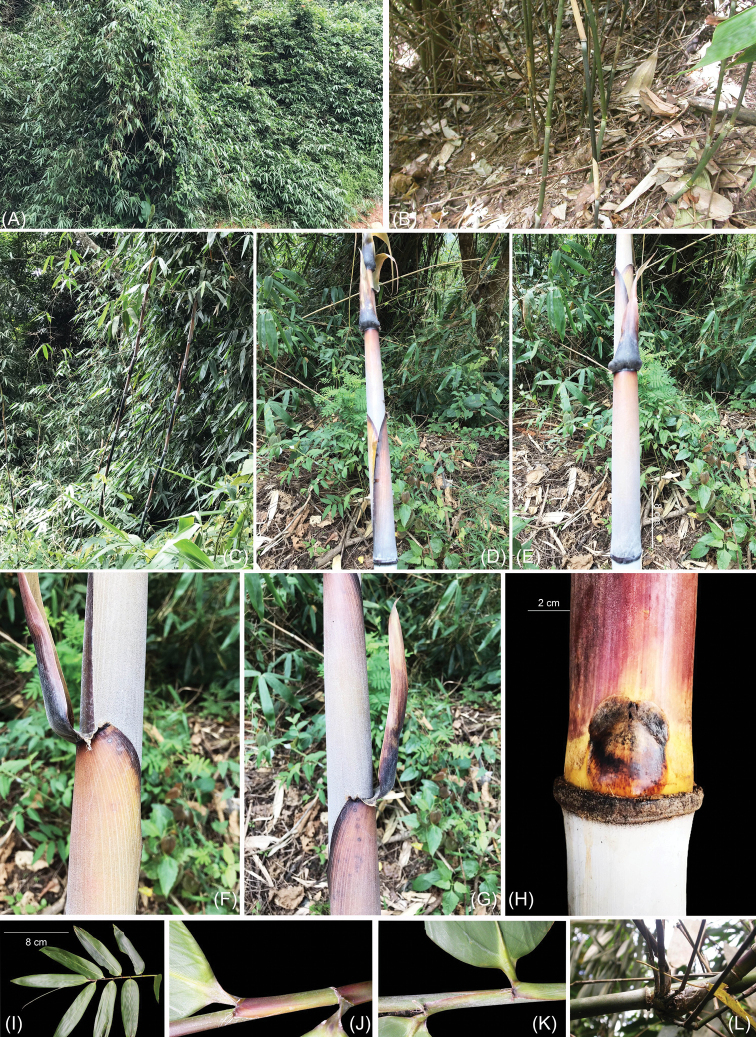
*Yersinochloanghiana* V.T.Tran: **A** habitat **B** clump **C, D** shoots **E** culm leaf (dorsal view) **F, G** auricles **H** culm leaf swollen at the base **I** leafy branch **J, K** section of leafy branch **L** branches several with middle one dominant. Photos by Tran Van Tien from type locality.

**Figure 2.**
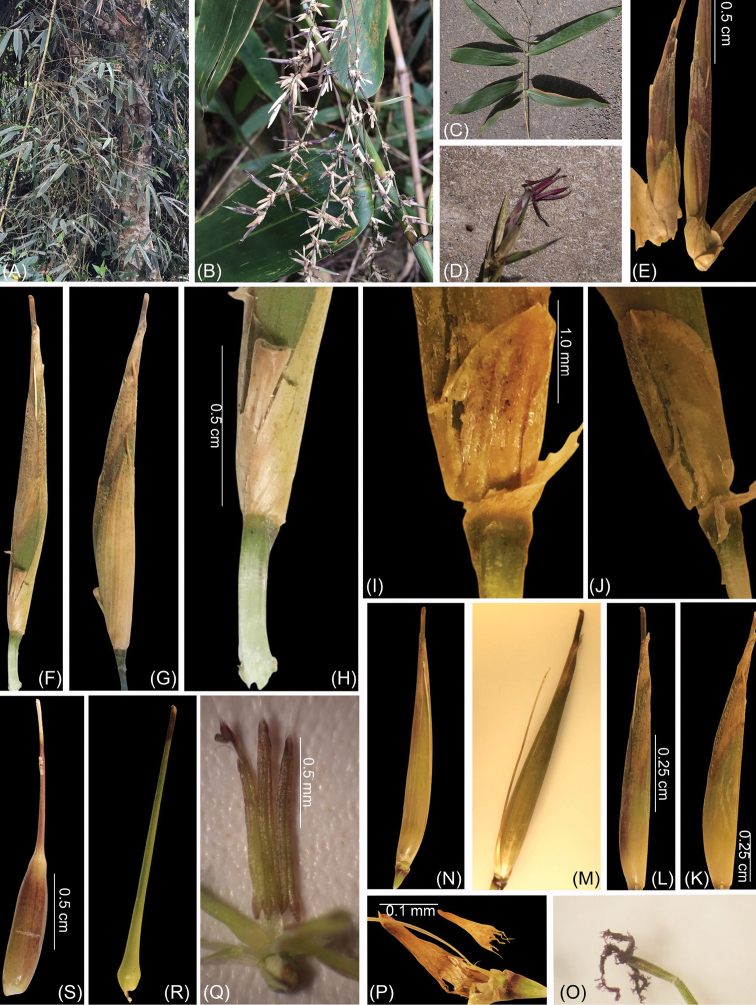
*Yersinochloanghiana* V.T.Tran: **A–C** inflorescence terminating at leafy branches **D, E** pseudo-spikelets **F, G** perfect florets **H** rachilla internode **I, J** prophyllate bud keeled **K** lemma **L** lemma (dorsal view) **M** palea with rachilla extension **N** palea (dorsal view) with rachilla extension **O** stigmas **P** lodicules **Q** stamens **R** caryopsis **S** fruit. Photos by Tran Van Tien from type locality.

**Figure 3.**
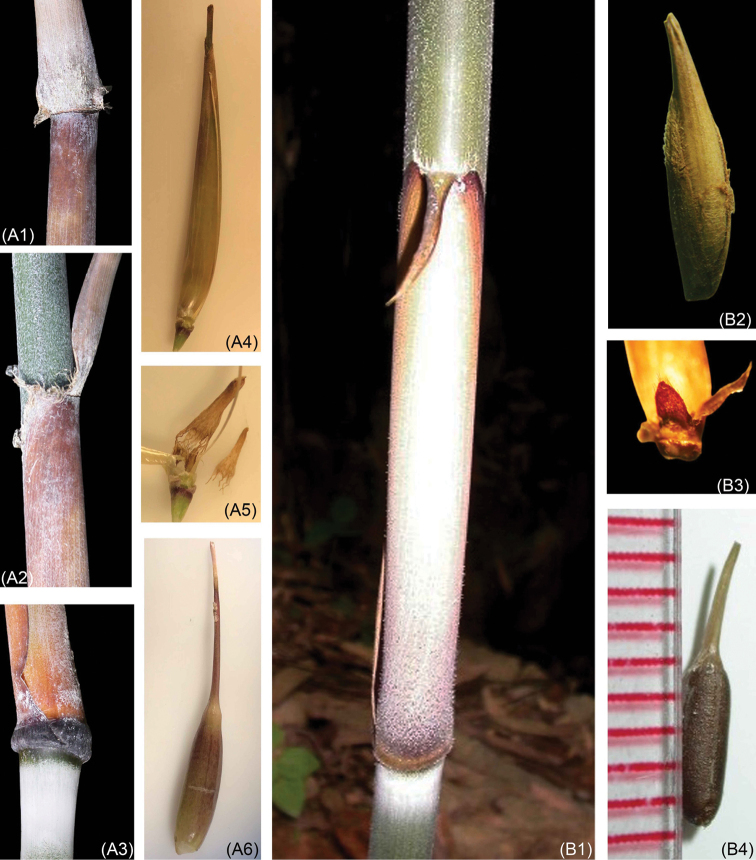
*Yersinochloanghiana* V.T.Tran and *Y.dalatensis* H.N. Nguyen & V.T. Tran: **A1–A3** culm leaves **A4** fertile floret with rachilla extension **A5** lodicules **A6** fruit **B1** culm leaf **B2** fertile floret without rachilla extension **B3** lodicules **B4** fruit. Photos by Tran Van Tien from type locality.

During our investigation of the bamboos from Braian Mountain, Di Linh District, Lam Dong Province, in southern Vietnam in December 2007, the authors found several populations of a clambering bamboo widespread and abundant through the degraded natural forest in valleys between 1100 and 1130 m a.s.l. Specimens of rhizomes, branches, culm leaves and flowers were collected and studied. We confirmed the presence of inflorescences terminating at leafy branches, pseudo-spikelets having only one perfect floret with no terminal vestigial flowers, the palea unkeeled and the anther apex bearing tiny spines, as was found in *Yersinochloadalatensis*. However, further detailed studies differentiated this specimen from the latter by characters having culm nodes with a thick swollen patella; culm leaf blades erect, swollen at the base; auricles conspicuous, margins bearing long hairs; palea dorsal view of palea showing rachilla extension and rudimentary floret at the apex; lodicules bifid at the base (Table 1, Figs 1–3). Besides that, the species is distinguished from *Cochinchinochloa*, because it has only one perfect floret, a terminal vestigial flower absent and anther apices bearing tiny spines. Its flower is typical in *Yersinochloa*. Otherwise, the culm leaf blades of *Cochinchinochloabraiana* have embraced the entire internode, auricles triangle-shaped, while the species have only half embraced the internode. Thus, it is not close to *Cochinchinochloabraiana*. These distinctive features indicate that this bamboo is readily diagnosed as a new species.

**Table 1.** Morphological comparisons of *Yersinochloanghiana* V.T.Tran, sp. nov. with *Y.dalatensis* H.N. Nguyen & V.T. Tran and *Cochinchinochloabraiana* H.N. Nguyen & V.T. Tran.

Characters		*Y.dalatensis*	*Y.nghiana*	*Cochinchinochloabraiana*
Internode		culm nodes uexhibit a thick swollen patella	culm nodes with a thick swollen patella	culms nodes with a thick swollen patella
Culm leaves	culm-leaves blade	reflexed, oblong	erect, swollen at the base, tardily deciduous, only half embraced the internode	erect, tardily deciduous, inflated at the base, embraced the entire internode
	auricles	absent	conspicuous	triangle shaped
Rachilla		absent	extension and a rudimentary floret at apex	extension bearing perfect and an imperfect floret elongate at maturity
Terminal vestigial flower		absent	absent	1
Perfect florets		1	1	2
Anther apices		bearing tiny spines	bearing tiny spines	absent tiny spines
Lodicules		3	3	3

## ﻿Materials and methods

This study is based on plant material collected from Braian Mountain, Di Linh District, Lam Dong Province, in southern Vietnam. The plant specimens were deposited at DLU, VNMN and VTN-Tay Nguyen Institute for Scientific Research. Vegetative parts were measured in the field; fresh flowers were examined under a light-microscope and colour photographs were taken using a camera. Other similar species were used for critical comparison.

## ﻿Taxonomic treatment

### ﻿ Yersinochloa nghiana

Taxon classificationPlantaePoalesPoaceae

V.T.Tran sp. nov.

082E73E2-32EA-5C74-9ED6-40C789B37AC1

urn:lsid:ipni.org:names:77317220-1

Figs 1 , 2

#### Diagnosis.

*Yersinnochloanghiana* is morphologically most similar to *Y.dalatensis* and *Cochinchinochloabraiana* with culms and branches scrambling or hanging over nearby vegetation or trees, lodicules 3, stamens 6, filaments free, caryopsis oblique, with a relatively thin pericarp. However, *Y.nghiana* is distinguished from *Y.dalatensis* by culm nodes with a thick swollen patella (vs. culm nodes without a thick swollen patella), culm-leaves blade erect (vs. reflexed), auricles conspicuous (vs. auricles absent). It also differs from *Cochinchinochloabraiana* in culm-leaves blade half embracing the internode (vs. culm-leaves blade embracing the entire internode), a terminal vestigial flower absent (vs. a terminal vestigal flower 1), perfect florets 1 (vs. perfect florets 2), anther apices bearing tiny spines (vs. anther absent tiny spines).

#### Type.

Vietnam. Lam Dong Province, Di Linh District, Brain Mountain, E, 1216 m a.s.l., 11°27'25"N, 108°3'41"E, 10 Sep 2022, *V. T. Tran DLU 0463* (holotype DLU!; isotype VNMN!, VTN!).

#### Description.

Culms and branches scrambling or hanging over nearby vegetation or trees, 5–10 m tall; internodes 40–80 cm long and 3.5–4.5 cm in diameter; when young, densely covered with appressed white hairs; culm walls 0.8–1.0 mm thick; nodes with a thick swollen patella. Branches several with middle one dominant, elongating. Culm leaves black-purplish, sheath with dense appressed white hairs on the abaxial surface; 25–28 cm long and 10–12 cm wide at base; apex 7–8 cm wide and truncate; margins bearing dense white-brown hairs; leaf blades erect, swollen at the base, tardily deciduous, purple-black, 20–25 × 4–5 cm, abaxially with dense white hairs at the base; auricles conspicuous, 2.0–2.2 × 0.2–0.3 cm; ligule short, ca. 1 mm, entire. Leafy branches bearing 5–6 leaves, foliage leafy branches distichously arranged along its length; foliage leaf blades wedge-shaped, 25–28 × 4.0–4.5 cm, acute or cuneate-obovoid at base, glabrous; veins 15–18 pairs; sheaths with ciliate margins, auricles with dense bristles 3–5 mm long; inner ligule a low rim, ca. 1 mm; pseudo-petiole ca. 5–6 mm length. Inflorescences terminating at leafy branches, indeterminate; pseudo-spikelets typically 2.2–2.0 cm long; each subtended by a prophyllate bud, keeled, with ciliate margins, 2.0–2.2 × 0.6–0.8 mm and consisting of one glume, one perfect floret. Rachilla internode below fertile floret ca. 0.5 cm. Fertile floret 1.0–1.2 × 0.2–0.4 cm; lemma oblong lanceolate, 1.0–1.2 × 0.5–0.6 cm, veins 7–9, at apex acute with 0.2 mm long, margins and adaxial side bearing dense white cilia; palea glabrous, distally obviously grooved, dorsal view showing rachilla extension and a rudimentary floret at apex, 1.0–1.2 × 0.5–0.6 cm, acute at apex, base spirally involute; lodicules 3, obovate or oblong, acute at apex, purple, ca. 0.2–0.3 × 0.1–0.2 mm, with ciliate margins, bifid at base. Stamens 6; filaments free, 1.0–1.2 cm; anther ca. 1 mm, purple, apices bearing 3 tiny spines, ca. 1 mm. Ovary green, glabrous with a long style, 1.2–1.0 cm; stigmas 3, purple; caryopsis oblique, with a relatively thin pericarp, 0.9 –1.0 × 0.1–0.2 cm, with a long style, ca. 1.3 cm.

#### Distribution and habitat.

*Yersinochloanghiana* grows in degraded natural forest in valleys, but is also common along rivers and valleys, between 1100 and 1130 m a.s.l., in Braian Mountain, Di Linh District, Lam Dong Province, Vietnam.

#### Phenology.

The plants were found flowering in December 2007. New shoots developed in June to August.

#### Local uses.

*Yersinochloanghiana* is of considerable importance to the local people. Its culms are used for making handicrafts and household tools.

#### Etymology.

The new species is named in honour of Dr. Nguyen Hoang Nghia for his contributions to the bamboo research in Vietnam.

#### Preliminary conservation status.

The species *Yersinochloanghiana* sp. nov. is only known from a single population in Braian Mountain, Di Linh District, Lam Dong Province, Vietnam. This single population has no more than 500 mature clumps, all growing in degraded natural forests in valleys, but is also common along rivers and valleys. According to IUCN Red List Categories and Criteria (IUCN 2022), the species is classified as data deficient (DD) and it needs more surveys.

## Supplementary Material

XML Treatment for Yersinochloa nghiana
